# Dynamics of human B and T cell adaptive immune responses to Kyasanur Forest disease virus infection

**DOI:** 10.1038/s41598-020-72205-1

**Published:** 2020-09-17

**Authors:** Santhosha Devadiga, Anita K. McElroy, Suresha G. Prabhu, Govindakarnavar Arunkumar

**Affiliations:** 1 Manipal Institute of Virology, Manipal Academy of Higher Education (Deemed To Be University), Manipal, Karnataka State 576104 India; 2 Department of Pediatrics, UPMC Children’s, Center for Vaccine Research, University of Pittsburgh, Pittsburgh, PA USA

**Keywords:** Immunology, Microbiology, Diseases, Medical research

## Abstract

Kyasanur Forest disease (KFD) is a tick-borne, acute, febrile viral illness endemic in southern India. No major studies have been done to understand the adaptive immune response during KFDV infection in humans. In this study, KFDV-positive patients were prospectively enrolled, and repeated peripheral blood collections were performed. Clinical and virologic characterization of these samples is reported along with phenotypic analysis of cellular immunity and quantitation of humoral immunity. We noted robust T and B cell responses, particularly of CD8 T cells, during KFDV infection in most of the patients. Virus clearance from the blood coincided with peak CD8 T cell activation and the appearance of KFDV-specific IgG. Increased frequency of plasmablasts and very few activated B cells were observed in the acute phase of KFD infection. Notably, only humoral immunity and activated B cell frequency in the acute phase correlated with prior KFDV vaccination, and only with 2 or more doses. This novel work has implications in KFD vaccine research as well as in understanding the pathogenesis.

## Introduction

Kyasanur Forest disease (KFD) is an emerging tick-borne viral disease known as “monkey fever” in the affected regions of southern India. Kyasanur Forest disease virus (KFDV) was first described in 1957 in Shimoga district, Karnataka (formerly Mysore) State, India^1^. Since 2012, several KFDV outbreaks have been documented in states along the Western Ghat region of India^2,3^. KFDV causes a severe febrile illness in humans and non-human primates. The estimated annual incidence of human KFD ranges from 100 to 900 cases, with overall case fatality rates of 2–5%^3,4^. Clinically, KFD presents with sudden onset of fever, chills, severe frontal headache, and generalized myalgia. Other symptoms include bradycardia, severe prostration, and conjunctival suffusion. Gastrointestinal distress and signs of hemorrhage, such as bleeding from mucosal membranes, have been noted^3^. Around 20% of KFD cases may develop neurologic manifestations after 2–3 weeks of illness, including meningismus and altered mental status, but rarely convulsion or loss of consciousness; no long-term sequelae are reported^3,5,6^.

Despite KFDV’s high pathogenicity in humans, very little is known about the magnitude or quality of the host immune response to infection. Currently, no specific anti-viral treatment is available for KFD. However, Karnataka State public health services, have been using a formalin-inactivated tissue culture-derived vaccine in KFD-endemic areas since 1990^2,7^, though reports on vaccine effectiveness are conflicting. A 2013 study demonstrated that one dose of a formalin-inactivated vaccine does not protect against KFDV infection, but that vaccine efficacy improved to 62.4% after 2 doses and 82.9% after 3 doses^8^. Additionally, vaccine-induced immunity is short-lived; a booster dose is recommended at 6–9 month intervals for 5 consecutive years after the last laboratory-confirmed case in monkeys or humans or identification of infected ticks in the area^7,8^. Such follow-up doses are difficult to achieve, thereby reducing actual effectiveness of the vaccine. However, no clear understanding exists of the humoral or adaptive immune correlates of protection following KFDV vaccination. Defining the immune correlates of protection will be important for developing more effective vaccines against KFDV.

To date, studies on the immune response against KFDV in humans have been limited to evaluating the innate immune response. A previous study reported elevated levels of circulating type 1interferon (IFN-I) in acute KFD patients that correlated with viremia^9^. Increased levels of pro-inflammatory cytokines, mainly IL-6, IL-10, IFN-alpha, and TNF-alpha, were noted during KFDV infection in the mouse model^10^. The present study focused on characterizing the frequency and magnitude of various peripheral lymphocyte phenotypes during the acute and convalescent phases of KFD illness in humans with or without a history of vaccination prior to KFDV infection.

## Methods

### Human subject research and biosafety precautions

Written informed consent was obtained from all patients enrolled in the study. The protocol was approved by the Institutional Ethical Committee of Manipal Academy of Higher Education (MAHEEC/003/2017). Specimen handling prior to inactivation was done in a biosafety level 3 laboratory with enhanced precautions, including wearing personal air-powered respirators, Tyvek suits, and double gloves. All the methods were performed following with the regional and national guidelines and regulations.

### Study design and setting

Peripheral EDTA blood samples were obtained from 44 patients with KFDV infection confirmed by KFDV-specific real-time PCR of serum. Patients were hospitalized at Sri Jayachamarajendra Government Hospital Thirthahalli (Karnataka), Community Health Centre Valpoi (Goa), Sub-District Hospital Sawanthwadi, or Rural Hospital Dodamarg (Maharashtra) in 2017 and 2018. Patients not willing to come for follow-up appointments and immuno-compromised patients were excluded. 85 blood samples were collected at the acute (days 1 to 14 post onset) and 39 at the convalescent (days 15 to 79 post onset) phase of KFD illness. Samples were transported under cold chain (2–8 °C) to Manipal Institute of Virology within 12 h of collection.

### Flow cytometry

T and B cell responses were measured using 8 different flow cytometry antibody panels (see Supplementary Table 1 for detailed information on antibody reagents). One panel was designed for absolute quantitation of total CD4 T, CD8 T, and B cells using Tru Count tubes [Becton Dickinson (BD), Franklin Lakes, New Jersey, USA]. Three panels were used to measure naive, central, and effector memory populations of CD4 and CD8 T cells and to determine the frequency of activated CD4 and CD8 T cells. One panel defined the frequency of plasmablasts and activated B cell populations. Three panels were used to evaluate CD8 T cells for proliferation, effector expression of cytotoxic molecules, and memory vs. naïve status. For phenotyping panels, 100–150 µL of whole blood was incubated with the surface stain antibody cocktail for 20 min at ambient temperature (AT) followed by incubation in FACSlyse (BD) and 2 phosphate buffered saline (PBS) washes prior to acquisition. For intracellular staining, cells were permeabilized using Cytofix/Cytoperm (BD) for 20 min at AT followed by 2 washes with 1 × perm/wash (BD), and then stained with intracellular antibodies for 20 min at AT. After 2 additional washes, data were acquired on an Accuri C6 flow cytometry analyzer (BD). Flow cytometry data were analyzed using Flowjo (Treestar, Ashland, Oregan, USA) software. Representative gating strategy was shown in supplementary Fig. 1.

### Humoral immune response to KFDV

Anti-KFDV IgM and IgG antibody titers were determined by ELISA using whole virus-infected cell lysates as antigen. Briefly, heat-inactivated (56 °C for 30 min) serum samples were tested in fourfold dilutions (1:100 to 1:6,400), and the assay was performed following the protocol of the Centers for Disease Control and Prevention (CDC, Atlanta, Georgia, USA). A sample was considered positive for IgM if the sum of the adjusted optical densities (OD) of all the dilutions was ≥ 0.45 and the titer was ≥ 400. Similarly, samples were considered positive for IgG if the sum for the adjusted OD of all dilutions was > 0.90 and the titer was ≥ 1:400 as reported by Ksiazek et al. 1999 with modifications^11^. Raw data of ELISA at different dilutions for the febrile and convalescent patients are provided in the supplementary Fig. 5.

### Real-time PCR for KFDV

RNA was isolated from plasma using a QIAmp Viral RNA Mini Kit (QIAGEN, Hilden, Germany) as per manufacturer’s instructions. Real-time PCR with KFDV-specific primers was performed as described elsewhere using the AgPath-ID One-Step reverse transcriptase PCR kit (Invitrogen, Carlsbad, California, USA) in a Quantstudio5 real-time thermal cycler (Applied Biosystems, Foster City, California, USA)^12^. Results were expressed as cycle threshold (Ct) value; this measurement of viral RNA is a correlate for viremia, virus isolation was not attempted.

### Data analysis

Data were recorded in MS Excel. Graphs were made using GraphPad Prism version 6.01 and statistical significance was determined by one-way ANOVA followed by Tukey`s multiple comparisons test or a Mann–Whitney test in the case of absolute acute versus convalescent cell numbers. Data on clinical, laboratory, and epidemiological information were obtained from the Acute Febrile Illness (AFI) surveillance study^13^. Vaccination status was self-reported.

## Results

### Demographic and clinical characteristics

Repeated samples were collected from a total of 44 KFD patients during acute and convalescent stages of the disease. Additionally, 12 healthy subjects who were not previously vaccinated and had no symptoms of clinical KFDV infection were included to establish the population normal range of cell phenotype distribution in the study population. Characteristics of the study population are summarized in Table 1. Mean age of the study population was 40.9 years. Mean times post symptom onset at which acute and convalescent samples were collected were 5.7 ± 2.7 and 35.7 ± 14.7 days, respectively. The most common clinical features were fever (reported by 100% of patients) followed by myalgia (88.6%), headache (84.1%), general weakness (84.1%), and nausea (59.1%). Relatively less common clinical features were vomiting (31.2%), diarrhea (27.3%), altered sensorium and/or seizures (6.8%), and hemorrhagic manifestations (2.3%). Of the 44 recruited patients with KFD, 50% received no KFDV vaccine, 50% had a history of at least one dose of KFDV vaccine, 33.2% had 2 doses received 15 days to 13 months before KFDV infection. Two (4.5%) of the infected patients died (Table 1). All the KFDV-infected patients were of low and middle socioeconomic strata, and 21 (48.8%) were agricultural laborers.

**Table 1:** Demographic, clinical, and laboratory characteristics of the KFD patients recruited in the study.

Variable	Mean (SD)	N (%)	H/O KFDV Vaccination		
			No (n = 22)	Yes (n = 22)	P value
**Mean age in year**	40.93 (13.1)				
**Gender**					
Male		18 (40.9)	11	7	0.3454
Female		26 (59.1)	11	15	0.4328
**Hospitals**					
CHC Valpoi, Goa		23 (52.3)	15	8	0.1444
JCH Thirthahalli, Karnataka		13 (29.5)	2	11	0.0125
RH Dodamarg, SDH Sawanthwad, PHC Banda Maharashtra		8 (18.2)	5	3	0.4795
**Socio economic status#**					
Low < 40		27 (61.3)	14	13	0.8474
Middle 40–70		17 (38.7)	8	9	0.8084
**Clinical status (n = 44)**					
Fever		44 (100)	22	22	1.0
Myalgia		39 (88.6)	18	22	0.108
General weakness		37 (84.1)	19	19	1.0
Headache		37 (84.1)	20	18	0.664
Nausea		26 (59.1)	12	15	0.537
Cough		20 (45.5)	9	12	0.547
Vomiting		14 (31.2)	10	5	0.203
Diarrhoea		12 (27.3)	7	5	0.736
Altered sensorium and/or seizure		3 (6.8)	3	0	0.233
Hemorrhagic signs		1 (2.3)	1	0	1.0
Hypotension		18 (40.9)	9	11	0.763
Deceased		2 (4.5)	2	0	0.488
**Laboratory features**					
Mean total leukocyte count/mm^3^ (n = 41)	2,588 (1627)		2,339	2,825	0.345
Mean platelet count × 10^3^/µl (n = 39)	124 (50)		104	139	0.031*
Aspartate aminotransferase IU/L (n = 25)	208.7 (338)		326	80	0.681
Alanine aminotransferase IU/L (n = 25)	102.6 (136)		147	54.6	0.907
Alkaline phosphatase IU/L (n = 23)	211 (92.9)		222.3	196.8	0.525
C-Reactive protein < 10 mg/dl (N = 24)	2.6 (2.2)		2.49	2.8	0.733
Mean duration hospitalization (days) (n = 43)	5.2 (2.7)		5.04	5.4	0.653
**H/O of KFD vaccination**					
Unvaccinated		22 (50)			
Vaccinated at least one dose		22 (50)			
Vaccinated two/three dose		16 (36.4)			

*Significant difference, #modified Udai Pareekh scale.

### Hematology and biochemical parameters

**Figure 1:**
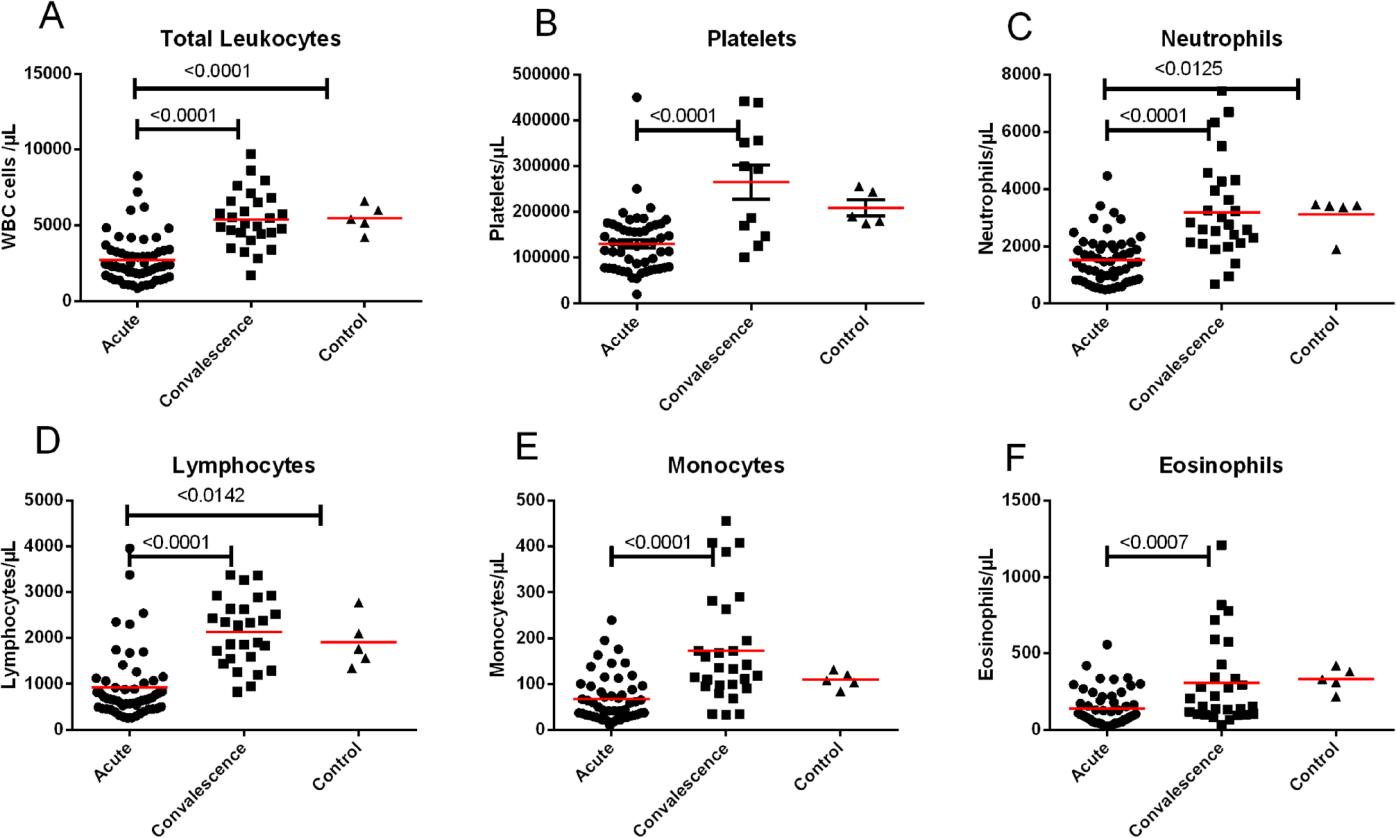
Kinetics of peripheral white blood cells and platelets during KFDV infection in humans. Line represents mean. Statistical significance was determined by one-way ANOVA followed by Tukey’s multiple comparisons test. Graphs were made using GraphPad Prism version 6.01.

The most common hematological abnormalities were marked leukopenia and thrombocytopenia. Overall leukocyte counts and platelet counts were low in all KFD patients (Fig. 1A,B). Marked leukopenia was observed in majority of KFD patients, whereas 62.9% of patients had thrombocytopenia in the acute stage. The median leukocyte count was 2,588 cells/µL (interquartile range (IQR): 1,727–3,250) in acute samples and 5,100 cells/µL (IQR: 4,440–6,600) in convalescent samples. Median platelet counts in acute and convalescent samples were 124,000/µL (IQR: 77,500–168,000) and 294,000/µL (IQR: 147,000–356,000), respectively. Peripheral leukocyte and platelet counts returned to normal during the convalescent phase of KFD. Peripheral leukocyte counts of some patients with severe KFD were below 1,000 cells/µL of blood. Peripheral neutrophil, lymphocyte, monocyte, and eosinophil counts markedly decreased during acute KFDV infection and normalized by the convalescent period (Fig. 1C–F). Liver enzymes such as aspartate aminotransferase (AST), alanine aminotransferase (ALT), and alkaline phosphatase (ALP) were moderately elevated during acute KFDV infection (Table 1).

### T cell responses during the acute and convalescent phases of KFDV infection

**Figure 2:**
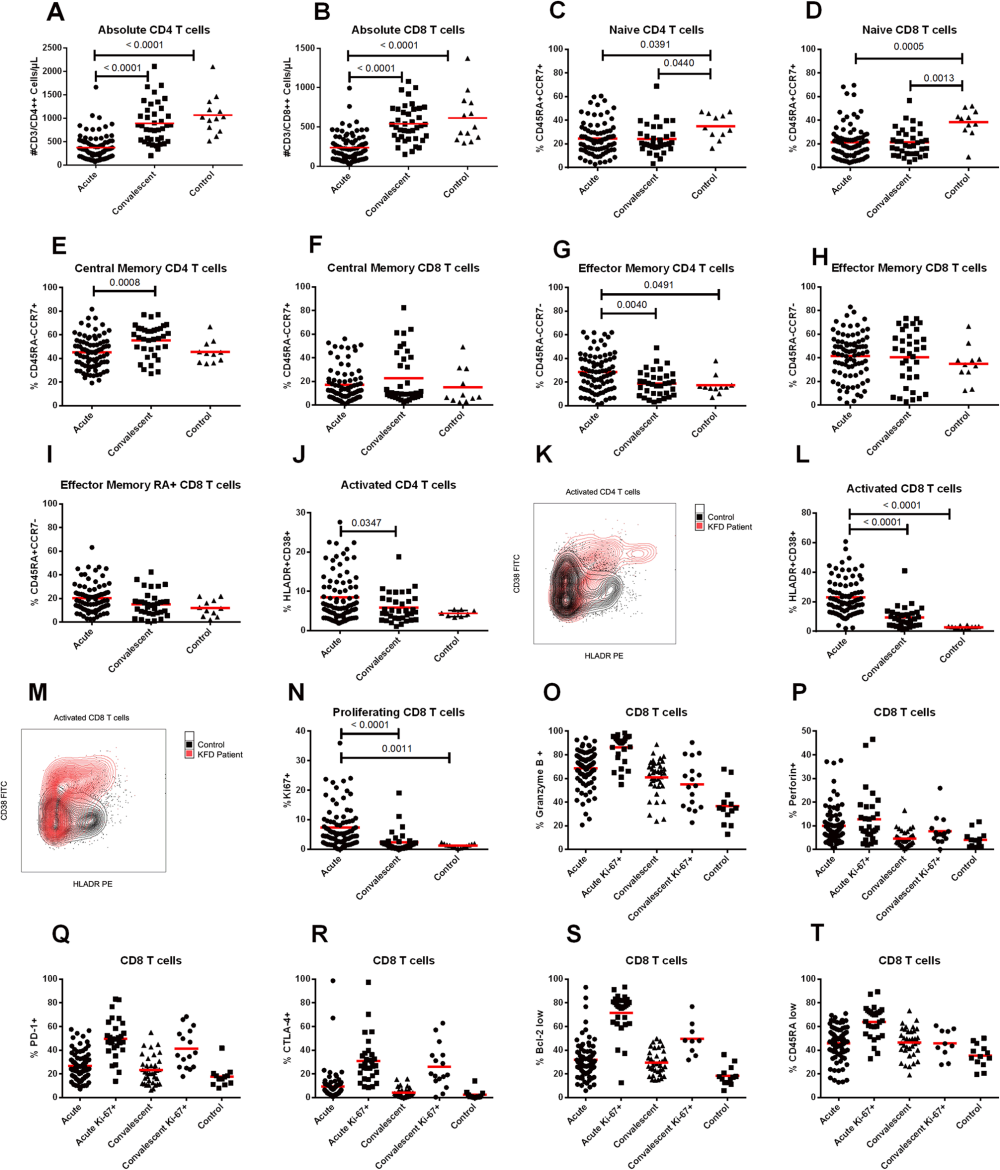
T cell responses during acute and convalescent KFDV infection. Absolute numbers of CD4 and CD8 T cells (**A**,**B**) (line refers to mean). Frequency of naïve, effector, and central memory CD4 and CD8 T cells (**C**–**I**). Representative flow plots for activated CD4 (**K**) and CD8 (**M**) T cells in infected patients versus a normal healthy control. Frequency of activated CD4 (**J**) and CD8 T cells (**L**). Frequency of proliferating CD8 T cells (**N**). CD8 + T cells and Ki67 + CD8 T cells expressing effector and memory molecules: granzyme B, perforin, PD1, CTLA-4, BCL-2, and CD45RA (**O**–**T**). Statistical significance was determined by one-way ANOVA followed by Tukey`s multiple comparisons test. Graphs were made using GraphPad Prism version 6.01.

Absolute numbers of CD4 and CD8 T cells were reduced in most of KFD patients during the acute phase of illness, consistent with the lymphopenia noted on routine hematology. Median absolute CD4 T cell numbers were 382 cells/µL of blood (IQR: 244–571) in acute samples and 899 cells/µL of blood (IQR: 817–1,232) in convalescent (Fig. 2A). Median absolute CD8 T cell numbers were 206 cells/µL (IQR: 106–344) in acute samples and 534 cells/µL (IQR: 376–730) in convalescent (Fig. 2B). The absolute number of CD4 and CD8 T cells returned to normal after the first week of illness in most patients, correlating with viral clearance from the blood. Phenotypic characterization of CD4 T cells showed a significant decrease in the frequency of naïve CD4 T cells and a concomitant increase in the frequency of effector memory CD4 T cells in acute and convalescent KFD samples in comparison to controls (Fig. 2C,G). Decreased frequency of central memory CD4 T cells was noted during the acute phase compared to convalescent phase (Fig. 2E). Phenotypic characterization of CD8 T cells showed a significant decrease in the frequency of naïve CD8 T cells, and a non-significant trend towards increased effector memory CD8 T cells in acute and convalescent KFDV infection in comparison to controls (Fig. 2D,H). No significant differences were noted in central memory CD8 T cells (Fig. 2F), or CD45RA + effector CD8 T cells (Fig. 2I).

Frequency of the activated T lymphocyte response was measured in all patients by analyzing co-expression of the activation markers HLA-DR and CD38 on CD4 and CD8 T cells. Representative flow plots depicting peak CD4 and CD8 T cell activation are shown (Fig. 2K,M). A significant increase in both activated CD4 and CD8 T cells was seen during acute KFDV infection compared to both healthy controls and convalescent patients (Fig. 2J,L). Increased frequencies of Ki-67 on CD8 T cells, indicating active proliferation, were also observed (Fig. 2N). There were no significant differences in T cell responses among vaccinated and unvaccinated KFD cases and also among two deceased cases (Supplementary Fig. 2, 3).

### Effector function characterization of CD8 T cells

CD8 T cells were further examined for expression of two markers of cytotoxicity (granzyme B and perforin), two inhibitory markers that are upregulated upon initial activation (PD-1 and CTLA-4), and CD45RA and Bcl-2, which would be low on effector T cells. The frequency of granzyme B + , perforin + , PD1 + , CTLA4 + , Bcl-2^low^, and CD45RA^low^ CD8 T cells, and Ki67 + CD8 T cells in acute and convalescent samples are depicted as compared to controls (Fig. 2O–T). Overall these data are consistent with an increase in effector CD8 T cells during the acute phase of KFD, with increased effector marker expression on cells that are also Ki-67 positive.

### B cell responses during the acute and convalescent phase of KFDV infection

**Figure 3:**
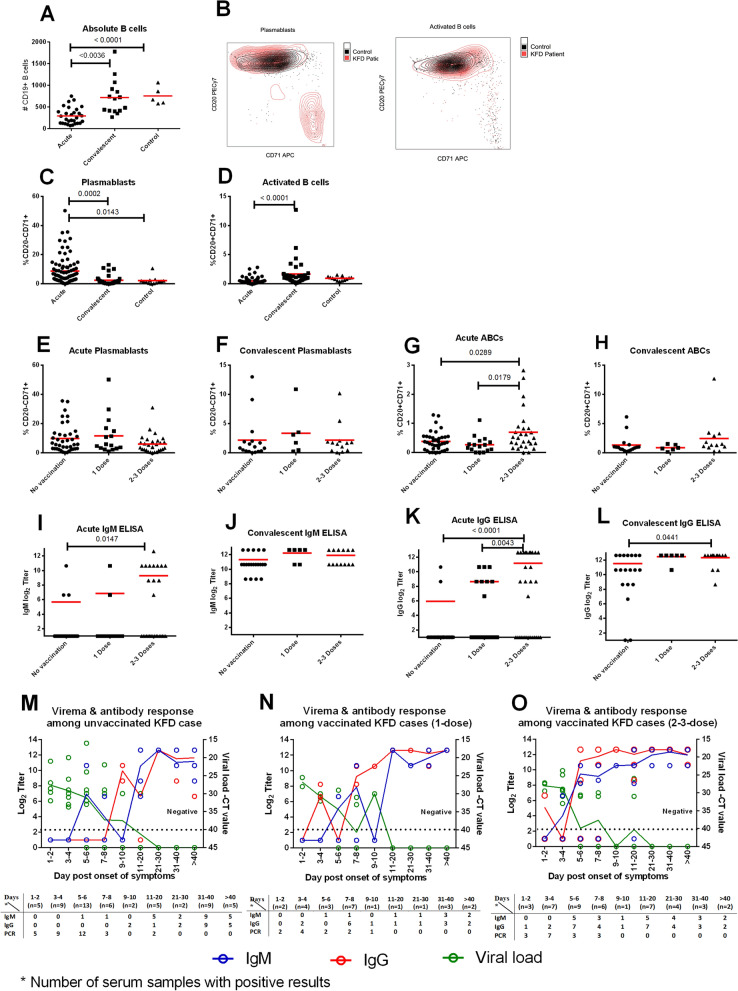
B cell responses during acute and convalescent KFDV infection and their relationship to prior vaccination. Absolute numbers of B cells (CD19 + lymphocytes) (**A**); line represents the mean. Representative flow plots for plasmablasts (CD20- CD71 +) and activated B cells (CD20 + CD71 +) in patients at the peak of the response versus a normal healthy control (**B**). Frequency of plasmablasts, activated B cells (**C**,**D**). Frequency of plasmablasts, activated B cells among vaccinated and unvaccinated patients at different phases of illness (**E**–**H**). IgM and IgG responses among vaccinated and unvaccinated patients at different phases of illness (**I**–**L**). KFDV-specific antibody responses and viral loads over time in vaccinated and unvaccinated patients (**M**–**O**). Dotted line represents the limit of KFDV detection by real-time PCR. Table under X-axis in figure **M**–**O** shows number of subjects at each time point. Statistical significance was determined by one-way ANOVA followed by Tukey’s multiple comparisons test. Graphs were made using GraphPad Prism version 6.01.

Lower numbers of absolute B cells were noted during acute KFD than convalescent, (n = 16) (Fig. 3A). Both plasmablasts (antibody-secreting cells) and activated B cells (ABCs) were measured using CD71 and CD20 markers on CD19 + B cells. Representative flow plots depicting peak plasmablast and ABC frequencies are shown (Fig. 3B). Increased frequencies of plasmablasts were observed in most KFD patients during the acute phase compared to both controls and convalescent patients (Fig. 3C). Very few ABCs were observed in the acute phase, but significantly increased during the convalescent phase of KFDV infection (Fig. 3D). Interestingly, no association was observed between vaccination status and plasmablast response (Fig. 3E,F). However, patients who had received 2 or 3 vaccine doses had higher percentages of ABCs during the acute phase, though not the convalescent (Fig. 3G,H).

### Humoral responses during the acute and convalescent phase of KFD infection

Antibody responses among vaccinated and unvaccinated KFD patients and correlation of these responses with viremia were evaluated. Individuals who received 2 or 3 doses of KFD vaccine had significantly higher acute IgM and IgG titers than did unvaccinated individuals or those who only received one vaccine dose, consistent with the expected rapidity and magnitude expected of a memory response (Fig. 3I–L). IgM response to KFDV was detectable as early as day 6 of illness and peaked 2–3 weeks after symptom onset. IgG response appeared on the 7th or 8th day of illness and peaked by the 2nd week. Virus clearance from the blood correlated with the appearance of KFDV-specific IgG (Fig. 3M–O).

### Viremia in KFD

KFDV was detectable in blood samples 1–12 days after symptom onset, with peak viremia on day 3 to 4 of illness. Cycle threshold (Ct) values ranged from 16–33. Clearance of viral RNA among patients who had received 2 doses of KFD vaccine occurred on average by day 6 of illness, although 2 of vaccinated patients were viremic up to 12 days post symptom onset (Fig. 3M–O). We did not find any significant difference in viremia periods observed among vaccinated KFD cases who developed IgG response very early in the course of infection (Supplementary Fig. 4).

## Discussion

This study reports a detailed analysis of longitudinal cellular and humoral immune responses to KFDV infection in humans. We noted robust T and B cell responses, particularly of CD8 T cells, during KFDV infection in most of the patients. Virus clearance from the blood coincided with peak CD8 T cell activation and the appearance of KFDV-specific IgG. Significant CD8 T cell activation was noted during the acute phase of KFD, as has been noted during infection or after vaccination with dengue, yellow fever, tick-borne encephalitis, Nipah, Ebola, and Lassa viruses, among others^14–19^. Activated (Ki67 +) CD8 T cells expressed high levels of acute effector markers PD-1 and CLTA-4 and cytotoxic molecules such as granzyme B and perforin, as well as low levels of Bcl-2, and CD45RA, suggesting that these effector cells were recruited from the naïve population via antigen-specific activation.

Interestingly, the magnitude of plasmablasts and ABCs noted in the acute phase of KFD was lower than reported for other acute viral infections in humans^20^. This may be secondary to the small window of time that these cells are present in the peripheral blood. Since most KFD patients were not sampled repeatedly during the acute phase, the window of maximal activation of these B cell populations may have been missed.

More than just an observational study of cellular immunity during KFD, this work provided a unique opportunity to assess how these various immune cell populations were modulated by prior vaccination. All patients in the study, both unvaccinated and vaccinated, became clinically ill after KFDV infection. One might hypothesize that prior vaccination would decrease the severity or limit the duration of the illness. Notably, the 2 patients who succumbed to the disease and the 3 patients who developed CNS signs did not receive KFDV vaccination, supporting the hypothesis that prior vaccination could limit the severity of the disease. Additionally, a statistically significant association was seen between the frequency of acute phase ABCs and IgM and IgG titers in patients who had received 2–3 doses of the vaccine, but not in those who had received a single dose. This is consistent with the previously reported poor efficacy of a single dose of KFDV vaccine and improved efficacy of additional doses. We might have missed low levels of anti-KFDV IgG as sensitivity of the assay with the cut off used needs to be further evaluated. Individuals who had received 2 or 3 vaccine doses also tended to clear KFDV sooner than unvaccinated individuals or those who received 1 dose, though some outliers with persistent viremia abrogated this effect.

The current KFDV vaccination strategy requires multiple doses of a formalin-inactivated preparation to achieve protection and annual boosting to maintain that protection. Since memory responses are more robust and develop faster than primary responses, the presence of a strong memory T cell response to KFDV antigens should have led to faster and more robust T cell responses in vaccine recipients than in unvaccinated persons, but this was not observed. The complete lack of statistically significant differences in T cell responses between vaccinated and unvaccinated individuals during the acute phase suggests that the current vaccine poorly primes T cells against KFDV. Therefore, one goal of future KFDV vaccine development efforts could be to target the generation of KFDV-specific T cell responses in addition to KFDV-specific antibody responses.

## Conclusion

This study describes the dynamics and phenotypic status of both B and T cell responses during KFDV infection in humans. The current KFDV vaccine has limited efficacy against KFDV infection, but may reduce the severity of KFD. A more effective KFDV vaccine is needed, and cellular and humoral responses after vaccination should be characterized in order to understand the correlation of protection, especially those related to KFDV-specific B and T cell responses. Towards this end, future studies will also be needed to develop and validate KFDV-specific reagents for use in studies of functional cellular immunity.

## Supplementary information

Supplementary information.
