# Supplementary material for: Dynamics of human B and T cell adaptive immune responses to Kyasanur Forest disease virus infection
Source: Sci Rep. 2020 Sep 17;10:15306. doi: 10.1038/s41598-020-72205-1 (PMC7499197; doi:10.1038/s41598-020-72205-1)
Supplement: Supplementary file 1 — Supplementary information. [file 41598_2020_72205_MOESM1_ESM.pdf]

Supplementary information

Dynamics of human B and T cell adaptive immune responses to Kyasanur Forest disease virus infection

Santhosha Devadiga[1]#, Anita K. McElroy[2,]#, Suresha G Prabhu[1], Govindakarnavar Arunkumar[1]@

Supplementary Figure 1: Representative gating strategy

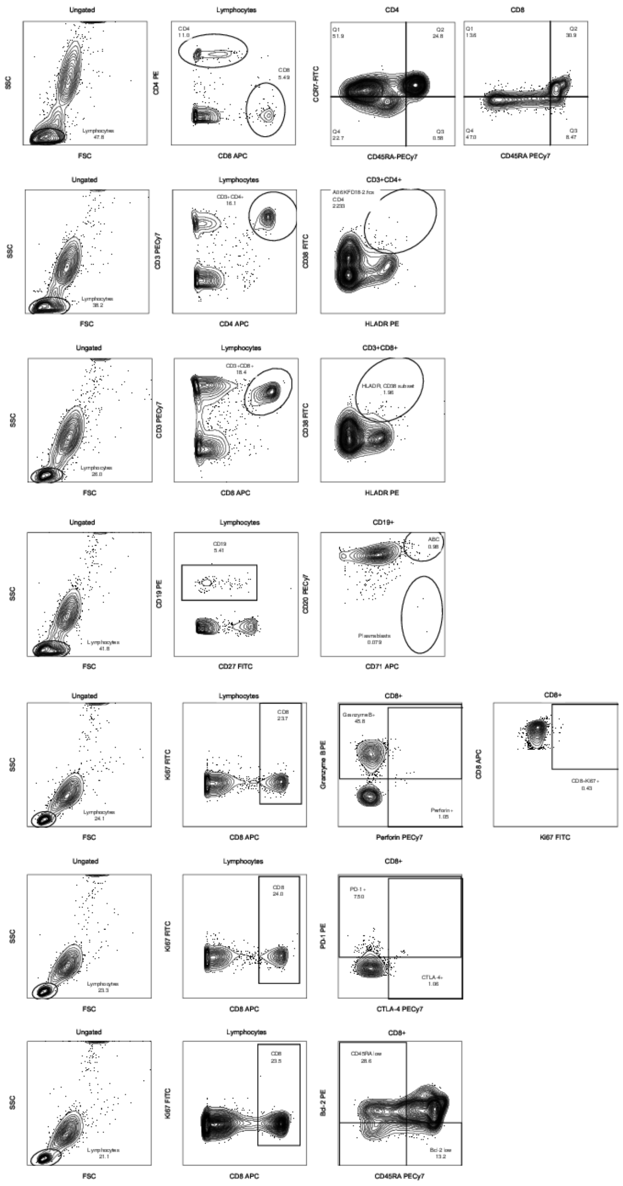

Suplementary figure 2:

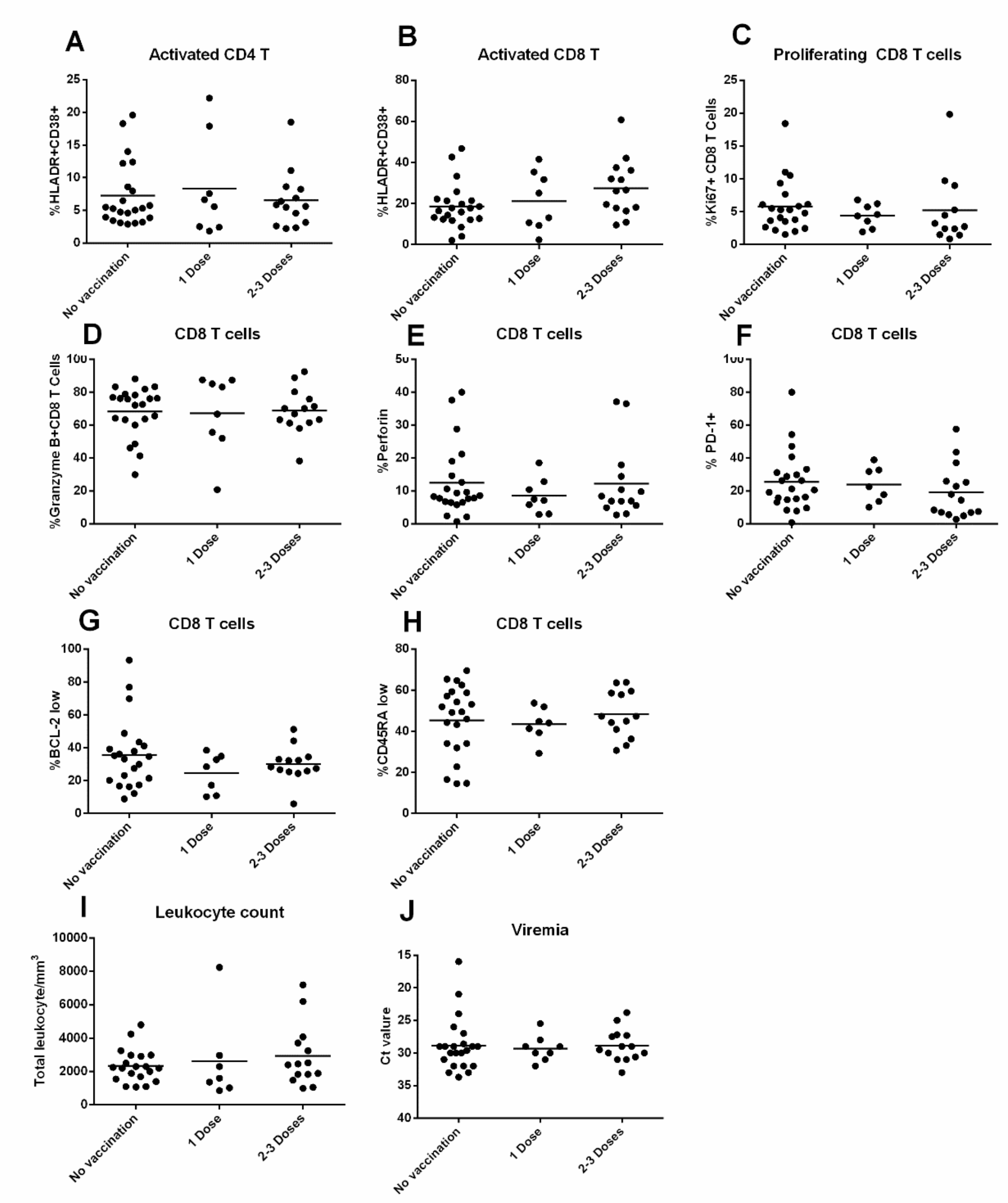

T cell response and viremia among vaccinated and unvaccinated KFD cases in the acute phase. (A, B) activated CD4 and CD8 T cell response among unvaccinated, vaccinated with 1 dose and 2-3 doses of KFDV vaccine. (C) Proliferating CD8 cells, (D-H) effector and memory T cell response. (I) total white blood counts among vaccinated and unvaccinated cases, (J) Acute

phase viremia. We did not find any statistically significant differences in T cell responses among vaccinated and unvaccinated KFDV cases. Mean is indicated by the line. Graphs were made using GraphPad Prism version 6.01

Supplementary figure 3:

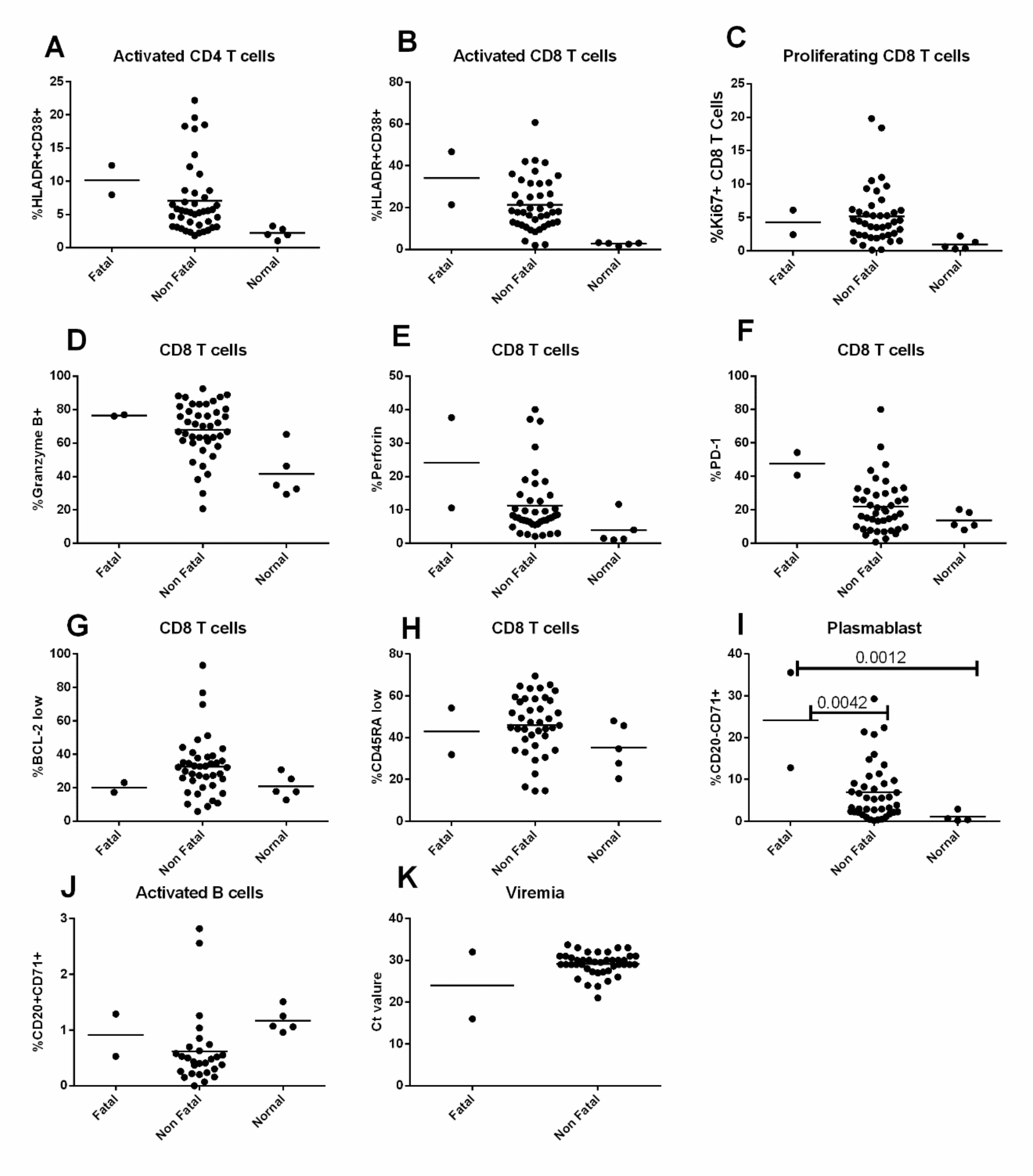

Cellular immune response and viremia among fatal and non-fatal KFD cases. Activated CD4 and CD8 T cell (A, B) proliferating CD8 cells (C), (D-H) effector and memory CD8 T cells, plasmablasts (I), activated B cells (J) and viral RNA load (K) in fatal and non-fatal KFD cases. Line indicates the mean. Graphs were made using GraphPad Prism version 6.01

Supplementary figure 4

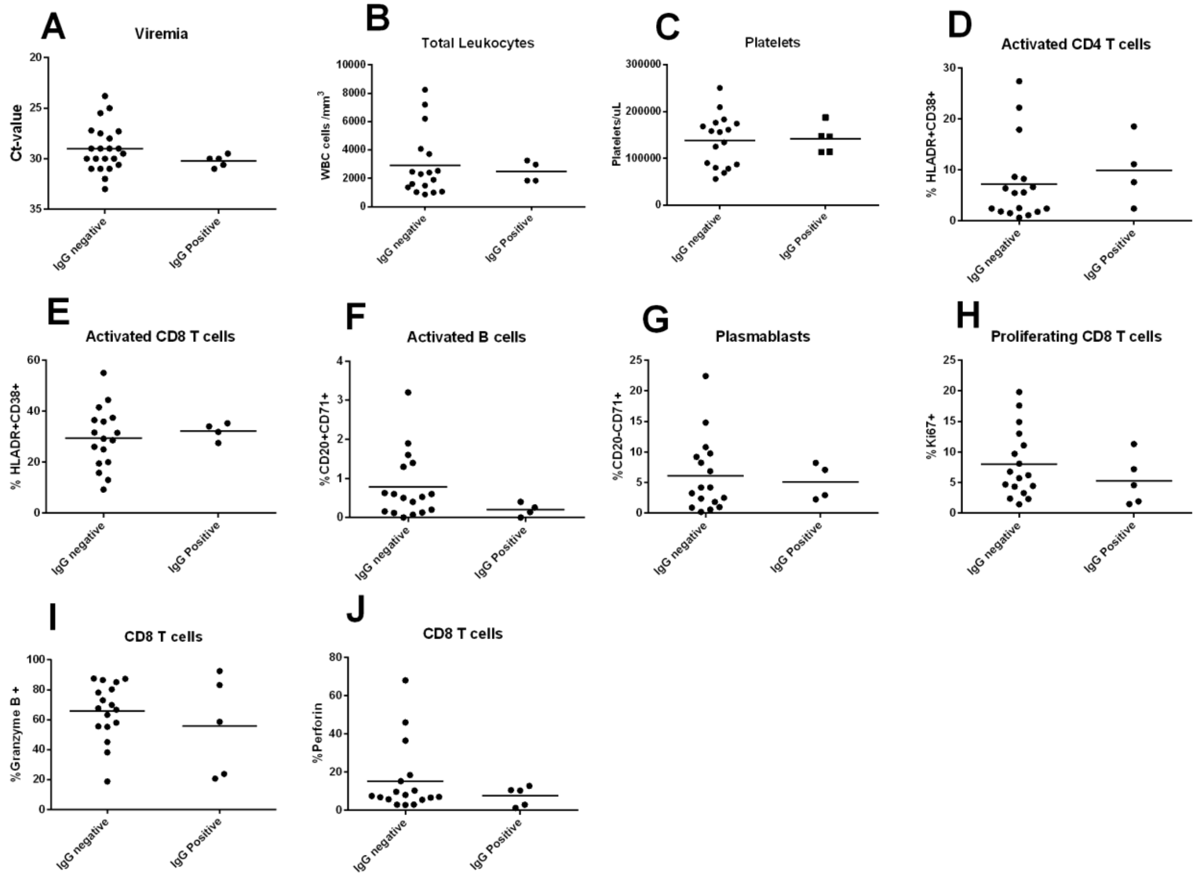

T and B lymphocyte response and viremia analysis among KFD vaccinated patients with and without IgG response at the first time point (first bleed): (A, B, C) comparison of viremia, total leukocyte and platelets, (D, E) activated CD4 and CD8 T cell response, (F, G) activated B cells and plasmablasts, (H) proliferating CD8 cells, (I,J) effector T cells response.

Line indicates the mean. Statistical significance was determined by one-way ANOVA followed by Mann-Whitney test. Graphs were made using GraphPad Prism version 6.01

.

Supplementary Figure 5

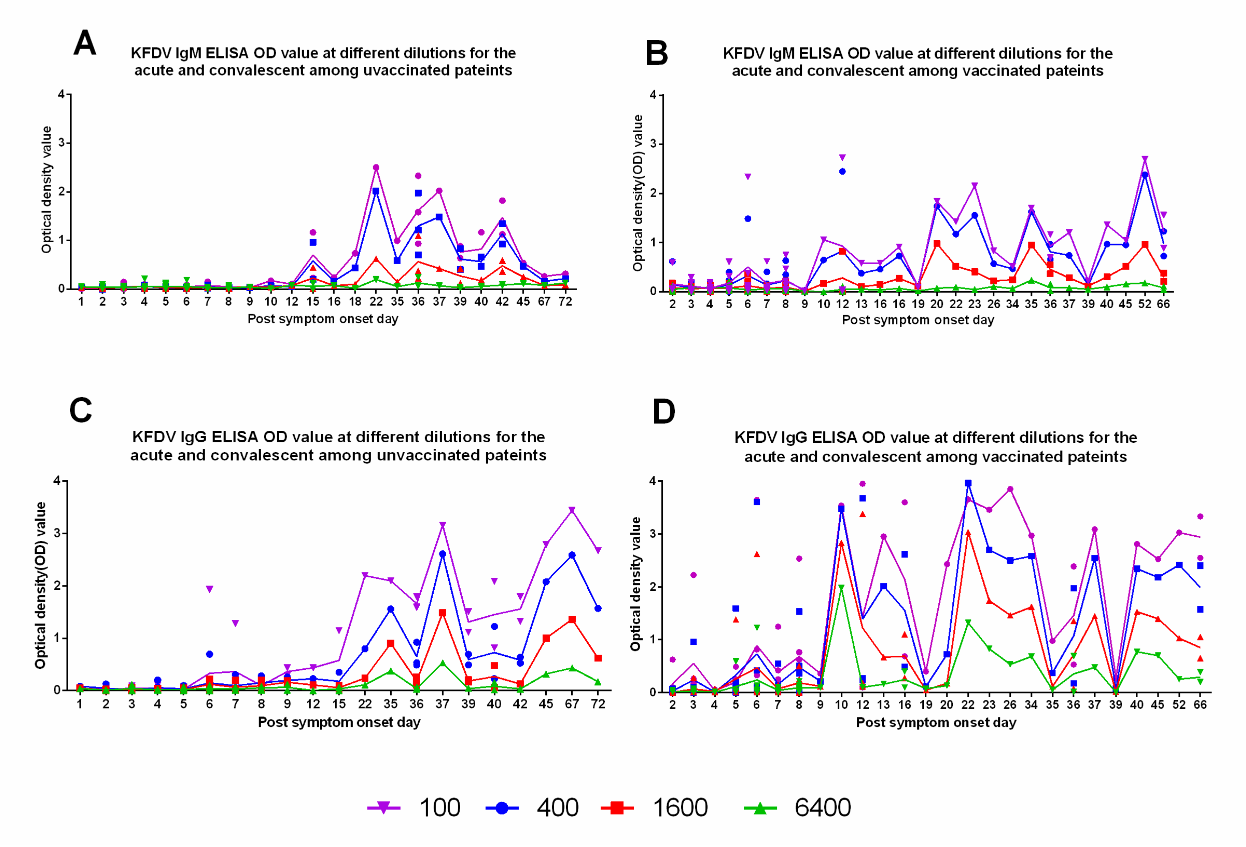

Raw data of ELISA curves at different dilutions (1:100, 1:400, 1:1600, 1:6400) for the acute and convalescent patients: KFDV IgM ELISA OD value of vaccinated and unvaccinated KFD individuals (A, B). KFDV IgG ELISA OD value of vaccinated and unvaccinated KFD individuals (C, D). Graphs were made using GraphPad Prism version 6.01

Supplementary Table 1: Antibody panel used for flow cytometry.

Panel	Target	Clone	Fluorophore	Company
True count	CD19	HIB19	FITC	BL
	CD45	H130	PE	BD
	CD3	SK7	PE/Cy7	BL
	CD8	RPA-T8	APC	BL
Naïve vs memory T cells	CD197 (CCR7)	3D12	FITC	BD
	CD4	RPA-T4	PE	BL
	CD45RA	HI100	PE/Cy7	BL
	CD8	RPA-T8	APC	BL
Activated CD4Tcells	CD38	HIT2	FITC	BL
	HLA-DR	G46-6	PE	BD
	CD3	SK7	PE/Cy7	BL
	CD4	RPA-T4	PE	BL
Activated CD8 T cells	CD38	HIT2	FITC	BL
	HLA-DR	G46-6	PE	BD
	CD3	SK7	PE/Cy7	BL
	CD8	RPA-T8	APC	BL
Plasmablast	CD19	HIB19	FITC	BL
	CD38	HIT2	PE	BL
	CD3, CD20	SK7, 2H7	PE/Cy5	BL,BD
	CD27	O323	APC	BL
Activated B cells	CD27	O323	FITC	BL
	CD19	HIB19	PE	BD
	CD20	2H7	PE/Cy7	BD
	CD71	Cy1G4	APC	BL
Effector CD8 T	Ki67	Ki67	488	BL
	Granzyme B	GB11	PE	BD
	Perforin	delta G9	PE/Cy7	BL
	CD8	RPA-T8	APC	BL
Effector CD8 T	Ki67	Ki67	488	BL
	PD-1	EH12.2H7	PE	BL
	CD152 (CTLA-4)	L3D10	PE-Cy7	BL
	CD8	RPA-T8	APC	BL
Effector CD8 T	Ki67	Ki67	488	BL
	Bcl-2	Bcl-2/100	PE	BD
	CD45RA	HI100	PE/Cy7	BL
	CD8	RPA-T8	APC	BL

BD, Becton-Dickinson Biosciences; BL, BioLegend.
